# Beyond adrenal fatigue: reframing the adrenal stress index through neutrophil-mediated glucocorticoid resistance

**DOI:** 10.3389/fendo.2026.1785454

**Published:** 2026-03-19

**Authors:** Giuseppe Cardillo

**Affiliations:** MEDYLAB S.r.l., Bologna, Italy

**Keywords:** corticosteroid-binding-globulin, salivary cortisol, salivary DHEAS, low-grade chronic inflammation, neutrophil-elastase

## Abstract

**Background:**

The Adrenal Stress Index (ASI) is widely used in functional medicine but dismissed by mainstream endocrinology. Traditionally interpreted as a measure of adrenal secretory capacity, it has been criticized for lacking clinical validity. Yet salivary cortisol and dehydroepiandrosterone sulfate (DHEAS) may offer more than redundant hormone monitoring: they may capture the organism’s ability to maintain resilience against chronic inflammation.

**Hypothesis:**

We propose that the ASI should be reframed not as a test of adrenal “fatigue,” but as a candidate biomarker of inflammatory adaptation. Cortisol, bound to corticosteroid–binding globulin (CBG), is released at inflamed sites through neutrophil elastase stored in azurophilic granules. Since these granules are formed only at the promyelocyte stage, their depletion under chronic demand leads to neutrophil exhaustion and impaired cortisol delivery. DHEAS, conversely, buffers cortisol’s catabolic and immunosuppressive actions and declines with age or persistent stress. The balance between cortisol exposure and DHEAS dynamics, therefore, reflects the efficiency of the HPA–immune–metabolic network rather than adrenal output alone.

**Supporting evidence:**

Flattened diurnal slopes, attenuated cortisol awakening response, evening–predominant DHEAS, and paradoxical elevations of salivary sex steroids under low cortisol/DHEAS conditions illustrate how chronic inflammation reprograms endocrine networks. These profiles align with clinical phenotypes such as frailty, sarcopenia, and metabolic dysfunction.

**Implications:**

Reframing the ASI as a measure of resilience highlights its potential as a research framework for investigating endocrine–immune adaptation. Standardized ELISA–based protocols, using fixed multi-timepoint daily sampling schemes, supported by LC–MS/MS validation, and the development of composite indices (e.g., cortisol AUC vs. DHEAS trapezoid gradient) may enhance reproducibility and predictive value. If validated in prospective cohorts, the ASI may provide a useful experimental tool for studying systemic adaptation in aging, chronic inflammation, and stress–related disorders.

**Conclusion:**

The ASI may provide a framework for investigating interactions between stress and inflammation. By bridging conventional endocrinology with functional perspectives, it may offer a conceptual framework for investigating inflammaging and biological resilience.

## 1 Introduction

Stress and inflammation are ubiquitous drivers of chronic disease, yet the clinical tools to assess the organism’s capacity to cope with these stressors remain a subject of intense debate. Among these tools, the Adrenal Stress Index (ASI) – a non-invasive salivary test measuring the diurnal rhythm of cortisol and dehydroepiandrosterone sulfate (DHEAS) – occupies a controversial niche (1, 2). The rationale for the ASI lies in the unique properties of saliva as a biological fluid. Unlike serum cortisol, which primarily reflects the total hormone concentration (largely bound to corticosteroid-binding globulin, CBG, and albumin), salivary cortisol correlates strongly with the free, biologically active fraction of the hormone (3). Since only the free hormone can passively diffuse into cells and bind to glucocorticoid receptors, salivary levels provide a superior readout of tissue-level glucocorticoid exposure compared to total serum levels, particularly in states where binding globulins are altered, such as insulin resistance, inflammation, or estrogen therapy (4). Furthermore, the non-invasive nature of saliva collection allows for repeated sampling, enabling the assessment of the circadian slope and the Cortisol Awakening Response (CAR), dynamic features that a single blood draw misses completely.

This dynamic resolution also underscores the limitations of urinary analysis, such as 24-hour urinary free cortisol or catecholamines. While urinary measures provide a useful integrated assessment of total daily hormone production, they fail to capture the critical circadian variations – the peaks and troughs – that characterize a healthy HPA axis response (5). Moreover, urinary catecholamines (epinephrine and norepinephrine) primarily reflect the cumulative sympathetic-adrenal-medullary (SAM) activity and are heavily influenced by renal clearance and total volume, whereas salivary cortisol offers a real-time, non-integrated proxy of the unbound steroid fraction (4, 6). By bypassing the “averaging” effect of urinary collection, the ASI provides a superior temporal resolution for identifying specific patterns of chronodisruption.

Despite these physiological advantages, a sharp dichotomy persists between mainstream endocrinology and functional medicine regarding the utility of the ASI. Mainstream endocrinology operates on a binary model of adrenal function: the gland is either competent or insufficient (Addison’s disease), hyperactive (Cushing’s syndrome) or normal. From this perspective, “sub-clinical” deviations in cortisol rhythm are often viewed as irrelevant noise. Consequently, the ASI is frequently dismissed as redundant because it does not add value to the diagnosis of overt adrenal pathology (7, 8).

Conversely, functional and integrative medicine practitioners rely on the ASI to detect “dysregulation” long before overt pathology appears. However, this approach has been hampered by the scientifically flawed nomenclature of “Adrenal Fatigue”. This term implies that the adrenal glands themselves become “tired” or depleted due to chronic stress – a concept that lacks histological and physiological evidence outside of autoimmunity or infarction (8).

The central hypothesis of this paper is that the conflict is one of interpretation, not measurement. The ASI is not a test of “adrenal secretory capacity” (which is indeed robust and rarely fails), but may reflect aspects of allostatic load and inflammatory resilience (9). When we measure salivary cortisol and DHEAS, we are not asking “Can the adrenals work?”, but rather “How is the neuro-endocrine-immune system adapting to the environment?”. By reframing the ASI away from the discredited “adrenal fatigue” model and towards a systems biology framework involving neutrophil function, CBG cleavage, and anabolic buffering, we propose a hypothesis-driven interpretive framework for investigating the biological cost of adaptation.

## 2 Cortisol bioavailability, neutrophils, and granulopoiesis

A critical limitation of conventional endocrine assessment is the assumption that systemic hormone levels linearly reflect tissue-level action. In the case of cortisol, this assumption is flawed. In circulation, cortisol is predominantly protein-bound, with approximately 90% sequestered by corticosteroid-binding globulin (CBG) or albumin (10). While this reservoir buffers plasma levels, it restricts the hormone’s immediate bioavailability.

The key to targeted anti–inflammatory action lies in the delivery mechanism mediated by neutrophils. At sites of inflammation, neutrophils secrete neutrophil elastase, a protease stored in azurophilic granules, which specifically cleaves the reactive center loop of CBG (10, 11). This cleavage induces a conformational change that reduces CBG’s affinity for cortisol by orders of magnitude, effectively “dumping” free cortisol precisely where it is needed to quench inflammation (10, 11).

Crucially, the enzymatic arsenal of neutrophils is finite. Azurophilic granules are synthesized exclusively during the promyelocyte stage of granulopoiesis in the bone marrow (12). Once a neutrophil enters circulation, it cannot replenish these granules (13). In acute inflammation, the bone marrow responds by releasing fresh waves of competent neutrophils. However, under conditions of chronic, low–grade inflammation (as seen in metabolic syndrome or chronic stress), the demand for elastase outstrips the maturation capacity of granulocytes. This leads to the release of “exhausted” or immature neutrophils that lack sufficient azurophilic granules (14).

**Figure 1:**
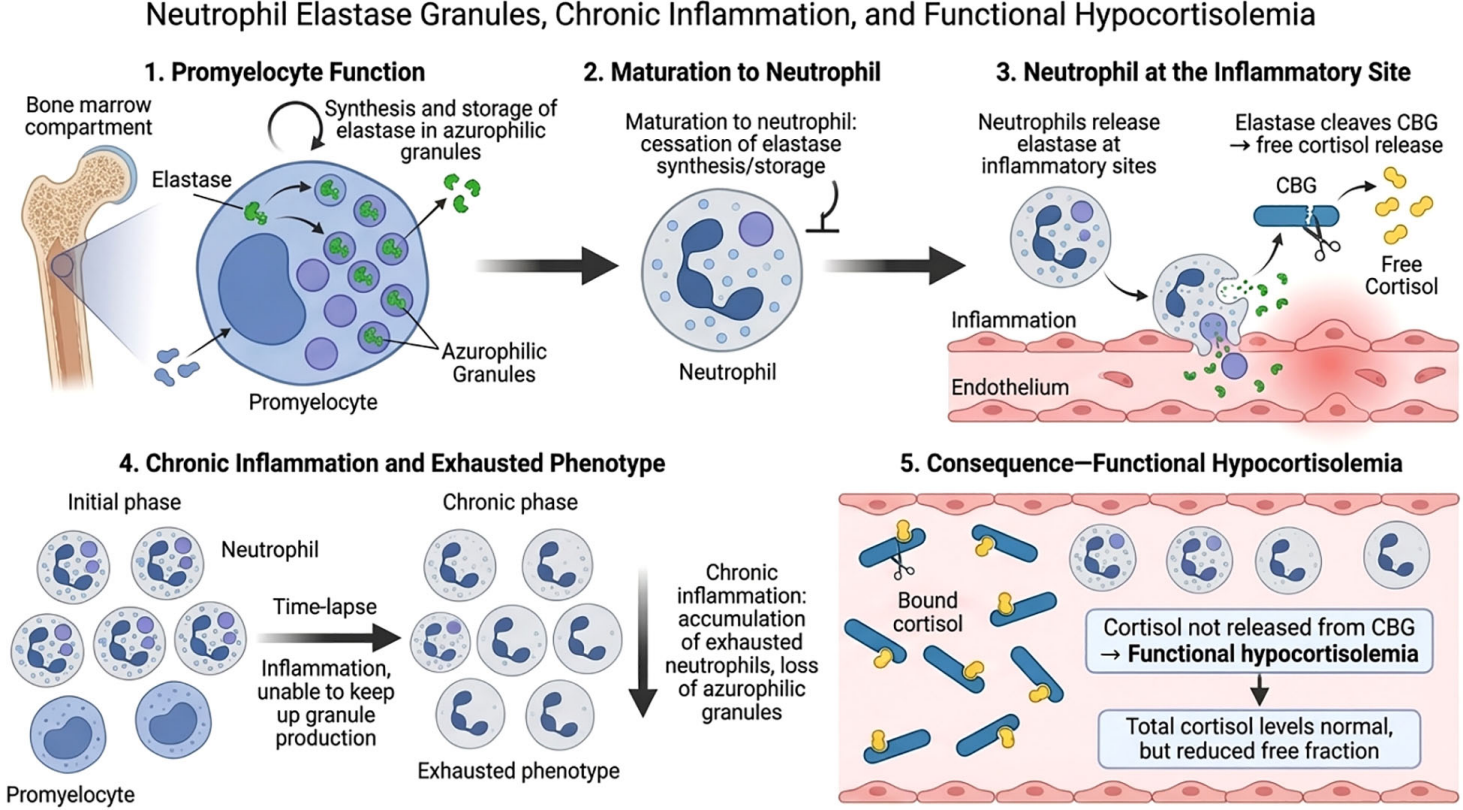
The Neutrophil–Elastase Hypothesis of Functional Hypocortisolemia. The diagram illustrates the proposed mechanism linking neutrophil exhaustion to localized glucocorticoid resistance. (1) Promyelocyte Function: In the bone marrow, neutrophil elastase is synthesized and stored exclusively during the promyelocyte stage. (2) Maturation: Mature neutrophils enter the circulation with a fixed, non-renewable supply of azurophilic granules containing elastase. (3) Normal Physiological Response: At the inflammatory site, neutrophils release elastase, which proteolytically cleaves Corticosteroid-Binding Globulin (CBG), thereby triggering the localized release of biologically active free cortisol from its carrier. (4) Chronic Inflammation and Exhausted Phenotype: Persistent systemic demand leads to a time-lapse depletion of the neutrophil granule pool. The “exhausted phenotype” consists of neutrophils depleted of elastase, unable to sustain the local delivery of cortisol. (5) Consequence – Functional Hypocortisolemia: Despite normal total serum cortisol levels, the lack of enzymatic cleavage results in cortisol remaining bound to CBG. This leads to tissue-level deficiency (functional hypocortisolemia) and impaired inflammatory resolution.

We propose that the clinical phenomenon often mislabeled as “adrenal fatigue” is, in many cases, a state of acquired local glucocorticoid resistance. The adrenal cortex may produce adequate cortisol (hence normal serum levels), but the delivery mechanism – neutrophil-mediated CBG cleavage – has failed due to granule depletion. The result is a paradox: systemic cortisol sufficiency coexistence with tissue-level glucocorticoid deficiency, leading to unremitting inflammation. The ASI, by measuring free salivary cortisol (which correlates with the unbound fraction), may offer a closer approximation of this bioavailable pool than total serum cortisol (**Figure 1**).

## 3 The role of DHEAS

Dehydroepiandrosterone sulfate (DHEAS), the most abundant circulating steroid in humans (15, 16), has long been dismissed as a mere precursor pool or a weak androgen. However, its physiological role is far more complex. Beyond its classical role as a steroid precursor, accumulating evidence indicates that DHEAS exerts direct immunomodulatory and neuroprotective effects (15–18). Experimental and clinical studies have shown that DHEAS can counterbalance glucocorticoid-induced immune suppression, modulate cytokine production, and preserve cellular immune competence under chronic stress conditions (17, 19). Moreover, age-related declines in DHEAS have been associated with increased inflammatory burden, frailty, and impaired stress resilience (20–22).

Recent integrative models of endocrine–immune interaction have emphasized the importance of considering cortisol and DHEAS not as independent variables, but as components of a coordinated adaptive system in which their dynamic balance reflects the organism’s capacity to maintain metabolic and inflammatory homeostasis. (17, 23, 24).

While cortisol mobilizes energy substrates to survive immediate threats (catabolism), DHEAS signals tissue repair, immune potentiation, and synaptic plasticity (anabolism). The molar ratio between cortisol and DHEAS is therefore a fundamental metric of the organism’s allostatic state. A decline in DHEAS is not merely a sign of aging (adrenopause); it is a biomarker of accelerated biological weathering.

Clinically, low DHEAS levels are consistently associated with sarcopenia, frailty, and cardiovascular mortality (20, 21). In the context of the ASI, DHEAS may serve as a proxy for the organism’s anabolic reserve (16, 25) within the context of integrated endocrine–immune adaptation (15, 17). Its measurement allows clinicians to distinguish between a stress response that is well-compensated (high cortisol, maintained DHEAS) and one that has become maladaptive (high/normal cortisol, depleted DHEAS), a distinction that serum cortisol alone cannot provide.

## 4 Integration: from static ratios to dynamic chronobiology

### 4.1 Standard sampling scheme and inferential scope

The standard ASI sampling scheme consists of four salivary cortisol measurements collected at fixed timepoints across the day (early morning, late morning, afternoon, and late evening), together with two DHEAS measurements obtained at the morning and late evening timepoints. This configuration is designed to capture the essential features of circadian organization, including total cortisol exposure (area under the curve), diurnal slope, and the temporal relationship between cortisol and its anabolic counter-regulator, DHEAS.

Importantly, the interpretive framework proposed here considers the ASI primarily as a descriptor of the current functional state of the HPA–immune axis and its circadian coordination, rather than as a fixed trait measure. In this context, day-to-day variability reflects physiological responsiveness to environmental, inflammatory, and metabolic conditions.

Multi-day sampling protocols may be used in research settings to improve estimation of intra-individual stability and trait-level characteristics. However, single-day profiles remain physiologically informative as state-level phenotypes, particularly when the objective is to characterize the system’s current adaptive configuration rather than its long-term baseline.

### 4.2 Circadian dynamics of cortisol secretion

Cortisol secretion follows a robust circadian rhythm characterized by low concentrations during the late night and a progressive increase toward the daytime active phase. This transition from the nocturnal nadir to higher daytime levels reflects coordinated activity of the hypothalamic–pituitary–adrenal (HPA) axis under circadian control, modulated by both central and peripheral regulatory mechanisms. Accordingly, cortisol secretion should be understood as a continuously evolving temporal process rather than as a series of discrete or isolated events.

The sampling schedule used in the ASI protocol captures key phases of this circadian trajectory, including early morning, late morning, afternoon, and late evening timepoints. These measurements do not attempt to isolate a specific awakening-related response, but instead provide a distributed representation of cortisol dynamics across the day. In this sense, the ASI profile may be viewed as an approximation of the integrated temporal pattern of bioavailable cortisol exposure, rather than a point estimate of instantaneous secretion.

This distinction is important because cortisol’s physiological effects depend not only on absolute concentrations but also on their timing, duration, and coordination with other endocrine and immune signals. Variations in circadian amplitude, slope, or phase relationships may therefore reflect differences in systemic regulatory organization rather than simple quantitative deficiency or excess.

Within the conceptual framework proposed here, the circadian structure of cortisol secretion represents one component of a broader adaptive system linking neuroendocrine signaling, inflammatory demand, and metabolic regulation. The ASI profile is thus interpreted not as a diagnostic measurement of adrenal output per se, but as a functional representation of circadian glucocorticoid availability within this integrated regulatory context.

Importantly, this interpretation should be regarded as a hypothesis-generating model intended to guide future longitudinal and mechanistic studies, rather than as an empirically validated clinical construct.

### 4.3 Mathematical formalization of the ASI profile

To further clarify the quantitative structure underlying the interpretive framework proposed here, the Adrenal Stress Index (ASI) profile can be expressed using a vector formulation that reflects the fixed temporal structure of the sampling protocol.

#### 4.3.1 Cortisol exposure and reference bound

Salivary cortisol concentrations measured at the four timepoints are represented as a column vector:


$$C=\left[\begin{array}{c} C_{1}\\ C_{2}\\ C_{3}\\ C_{4} \end{array}\right]$$

where each component represents the cortisol concentration (nmol/L) at the corresponding sampling time. Because the sampling intervals are fixed, total daily cortisol exposure can be computed using trapezoidal integration. This operation can be expressed equivalently in vector form as:


$$AUC_{C}=\frac{1}{2}w^{T}C$$

where w^T^ denotes the transpose of the weighting vector:


$$w=\left[\begin{array}{c} \Delta t_{12}\\ \Delta t_{12}+\Delta t_{23}\\ \Delta t_{23}+\Delta t_{34}\\ \Delta t_{34} \end{array}\right]$$

For the present sampling protocol, where the intervals are 4 h, 5 h, and 7 h, this becomes:


$$w=\left[\begin{array}{c} 4\\ 9\\ 12\\ 7 \end{array}\right]$$

and therefore:


$$AUC_{C}=\frac{1}{2}\left(4C_{1}+9C_{2}+12C_{3}+7C_{4}\right)$$

This vector formulation is mathematically identical to trapezoidal integration of the circadian cortisol curve and makes explicit that the ASI profile represents a weighted temporal integration of hormone concentrations determined solely by the sampling schedule.

Physiological reference limits for salivary cortisol at the four sampling timepoints are:


$$C_{min}=\left[\begin{array}{c} 13\\ 5\\ 3\\ 1 \end{array}\right]\;C_{max}=\left[\begin{array}{c} 24\\ 10\\ 8\\ 4 \end{array}\right]$$

Accordingly:


$$AUC_{C}^{min}=\frac{1}{2}w^{T}C_{min}=70\frac{nmol\cdot h}{L}\;AUC_{C}^{max}=\frac{1}{2}w^{T}C_{max}=155\frac{nmol\cdot h}{L}$$

#### 4.3.2 DHEAS exposure and reference bound

Salivary DHEAS concentrations measured at the morning and evening timepoints are denoted D1​ and D4​.

Given the fixed 16-hour interval:


$$AUC_{D}=\frac{16}{2}\left(D_{1}+D_{4}\right)=8\left(D_{1}+D_{4}\right)$$

DHEAS reference limits depend on sex and age. Let L_D_​ and U_D_​ denote the lower and upper physiological reference limits (nmol/L) appropriate for males:


$$L_{M}\left(age\right)=\left\{\begin{array}{cc} 22.8 & 18\leq age<31\\ 16.6 & 31\leq age<46\\ 10.6 & 46\leq age<61\\ 6.5 & age\geq 61 \end{array}\right\}\;U_{M}\left(age\right)=\left\{\begin{array}{cc} 62.4 & 18\leq age<31\\ 48.3 & 31\leq age<46\\ 31.2 & 46\leq age<61\\ 20.4 & age\geq 61 \end{array}\right\}$$

and for females:


$$L_{F}\left(age\right)=\left\{\begin{array}{cc} 17.4 & 18\leq age<31\\ 10.6 & 31\leq age<46\\ 7.3 & 46\leq age<61\\ 5.2 & age\geq 61 \end{array}\right\}\;U_{F}\left(age\right)=\left\{\begin{array}{cc} 50.5 & 18\leq age<31\\ 30.9 & 31\leq age<46\\ 21.7 & 46\leq age<61\\ 15.2 & age\geq 61 \end{array}\right\}$$

The corresponding minimum and maximum physiological exposures are:


$$AUC_{D}^{min}=16L_{D}\;AUC_{D}^{max}=16U_{D}$$

#### 4.3.3 Normalized exposure indices

To facilitate interpretation relative to physiological reference limits, normalized exposure indices are defined:


$$S_{C}=\frac{AUC_{C}-AUC_{C}^{min}}{AUC_{C}^{max}-AUC_{C}^{min}}=\frac{AUC_{C}-70}{155-70}=\frac{AUC_{C}-70}{85}\;S_{D}=\frac{AUC_{D}-AUC_{D}^{min}}{AUC_{D}^{max}-AUC_{D}^{min}}$$

These dimensionless parameters represent the position of the individual profile within the physiological reference envelope. By construction, S_C_ and S_D_ are dimensionless indices with S=0 corresponding to the lower reference envelope and S = 1 to the upper reference envelope; values below 0 or above 1 indicate profiles lying below or above the physiological reference bounds, respectively.

#### 4.3.4 Functional balance and circadian coordination

The balance between glucocorticoid and anabolic signaling can then be expressed as:


$$R_{CD}=ln\left(\frac{AUC_{C}}{AUC_{D}+\delta }\right)$$

Circadian amplitude is approximated by:


$$CDI=ln\left(\frac{C_{1}+\epsilon }{C_{4}+\epsilon }\right)$$

Temporal irregularity is quantified as:


$$VI=\frac{\left|C_{2}-C_{1}\right|+\left|C_{3}-C_{2}\right|+\left|C_{4}-C_{3}\right|}{mean\left(C\right)+\eta }$$

To ensure numerical stability near analytical detection limits, fixed stabilization constants were introduced based on assay lower limits of quantification (LLOQ).

For salivary cortisol:


ϵ=η=0,0262nmol/L

For DHEAS, the stabilization constant was defined as the area corresponding to a constant concentration equal to half the DHEAS LLOQ over the 16-hour sampling interval:


$$\delta =\left(\frac{0,1357}{2}\right)\times 16=1.086\frac{nmol\cdot h}{L}$$

These constants are fixed *a priori* based solely on analytical characteristics and do not materially affect interpretation within physiological ranges.

#### 4.3.5 Conceptual interpretation

These mathematical descriptors do not constitute a diagnostic algorithm but provide a quantitative representation of the functional relationships captured by the ASI profile, integrating total glucocorticoid exposure, anabolic buffering capacity, circadian coordination, and temporal organization.

This formalization demonstrates that the ASI profile represents a weighted integration of circadian endocrine dynamics rather than a static hormone measurement, and provides a quantitative framework for hypothesis-driven investigation of adaptive and maladaptive stress physiology.

## 5 Salivary profiles as putative phenotypes of inflammatory adaptation

When viewed through the framework proposed here, ASI patterns can be described as functional configurations of circadian glucocorticoid availability and anabolic buffering rather than as evidence of glandular “fatigue”. Importantly, the associations reported in the literature between salivary patterns and clinical or immune-related features are heterogeneous; therefore, the profiles below are presented as descriptive research phenotypes intended to guide empirical testing.

**Figure 2:**
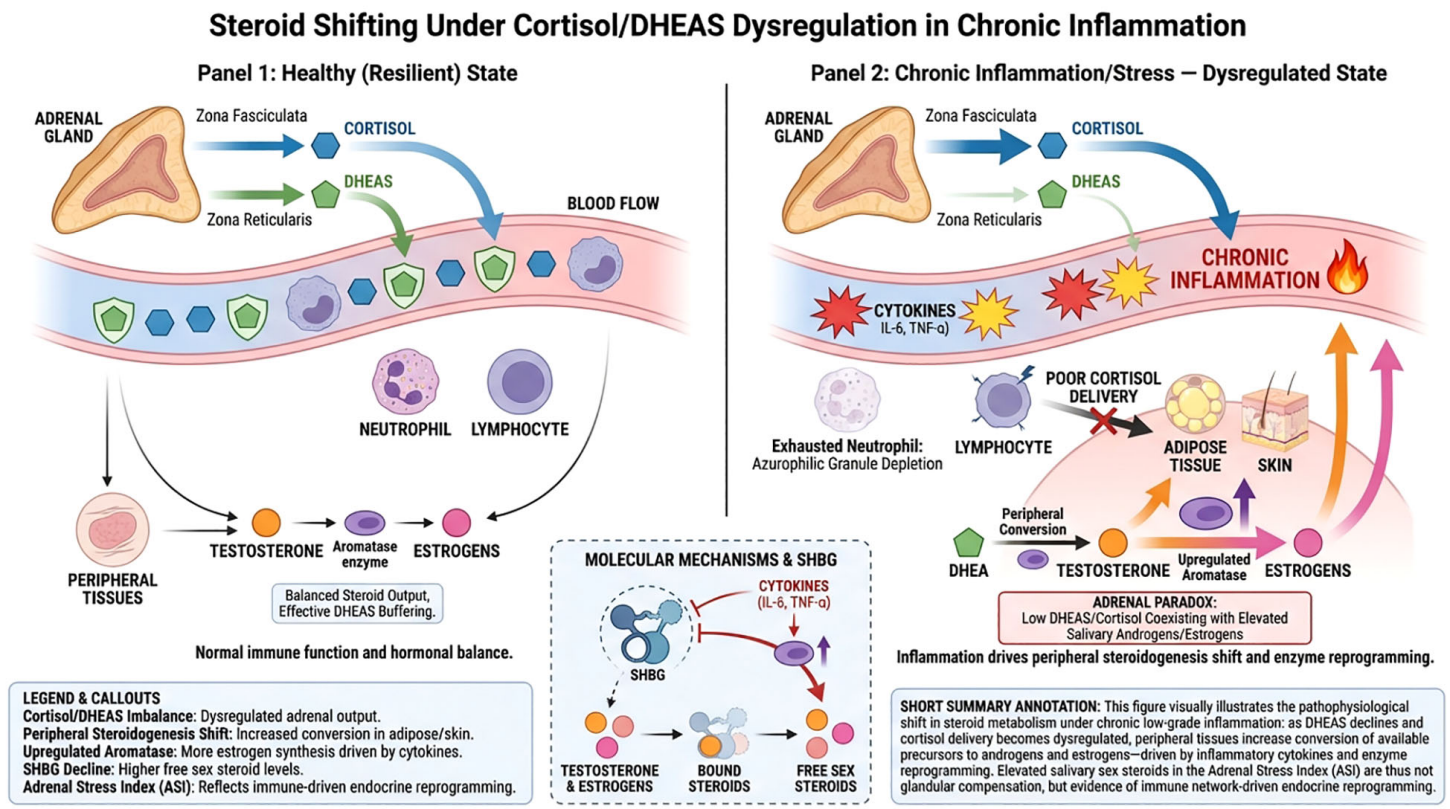
Steroid Shifting and the “Adrenal Paradox” in Chronic Inflammation. The diagram contrasts hormonal homeostasis with inflammatory dysregulation. Panel 1 (Healthy State): Balanced adrenal output of cortisol and DHEAS maintains systemic resilience and effective tissue delivery. Panel 2 (Dysregulated State): Pro–inflammatory cytokines (IL-6, TNF-a) impair central HPA control and deplete neutrophil granules, while simultaneously upregulating peripheral enzymes like aromatase. Molecular Mechanisms: Cytokine-driven suppression of SHBG increases the biologically active free fraction of sex steroids. This results in the “Adrenal Paradox” - the coexistence of low adrenal precursors with elevated salivary androgens/estrogens - reflecting immune-driven endocrine reprogramming rather than glandular failure.

1. Reduced early-morning rise. A reduced increase between early morning and late morning has been reported in several stress-related and inflammatory contexts. In this framework, it is treated as a descriptive marker of altered circadian organization rather than as a direct measure of adrenal secretory capacity. Its physiological meaning likely depends on sampling design, timing, sleep-related factors, and context-specific state variability (26, 27).
2. Flattened diurnal decline. A profile with relatively elevated late-evening cortisol or a reduced day–night contrast has been associated with inflammatory markers and adverse outcomes in some cohorts (1, 28). Within the present framework, this pattern is interpreted as reduced circadian amplitude and altered temporal organization, which may be relevant to endocrine–immune coordination but requires longitudinal clarification.
3. Low DHEAS buffering. Lower DHEAS concentrations, particularly when combined with higher integrated cortisol exposure, may indicate reduced anabolic buffering capacity (20, 21). Here, this is framed as a functional balance feature (catabolic–anabolic mismatch) that can be operationalized for research stratification rather than a clinical stage.
4. “Adrenal paradox” patterns. The coexistence of low adrenal precursors with relatively elevated salivary sex steroids has been described in inflammatory and metabolic contexts (29) and may reflect peripheral metabolism and binding-protein effects (**Figure 2**). In this manuscript, such patterns are treated as candidate signatures of immune–endocrine reprogramming that warrant mechanistic investigation.

Overall, these profiles are not proposed as diagnostic categories but as hypothesis-generating phenotypes that may help structure future mechanistic and longitudinal studies at the interface of circadian endocrinology, inflammation, and stress physiology.

## 6 Discussion: reframing the clinical utility of the ASI

The findings and the mechanistic framework presented here suggest that the clinical utility of the Adrenal Stress Index (ASI) has been historically misunderstood due to a reductionist view of the endocrine system. Conventional endocrine assessment primarily focuses on systemic hormone concentrations, whereas the ASI, by capturing the free fraction and the circadian rhythm, may offer a dynamic readout related to allostatic load and the efficiency of the cortisol delivery system.

### 6.1 The paradox of systemic sufficiency and local deficiency

The core of our hypothesis addresses a fundamental paradox in modern clinical practice: why patients with clear symptoms of glucocorticoid deficiency – such as unremitting fatigue, widespread pain, and chronic inflammation – frequently present with normal total serum cortisol. We propose that this discrepancy is not an analytical error but a physiological reality driven by neutrophil dynamics.

As discussed, the bioavailability of cortisol at the site of inflammation is an active, enzyme–mediated event. The cleavage of Corticosteroid-Binding Globulin (CBG) by neutrophil elastase is the “molecular switch” that releases free, biologically active cortisol precisely within the inflammatory micro-environment (10, 11). In the original framework of “adrenal fatigue”, the failure was attributed to the gland. Our hypothesis shifts the focus to the effector cells. Since azurophilic granules – the sole reservoir of neutrophil elastase – are synthesized only during the promyelocyte stage in the bone marrow and cannot be replenished (13), chronic inflammatory demand inevitably leads to a pool of “exhausted” neutrophils.

This may contribute to a state consistent with acquired local glucocorticoid resistance: the adrenal cortex may produce adequate cortisol (explaining normal serum levels), but the delivery mechanism is broken. The ASI, by measuring the free salivary fraction, may provide a closer approximation of the hormone pool that has escaped CBG binding, thus offering a functional window into this delivery efficiency that serum tests may miss.

### 6.2 DHEAS as the anabolic anchor and the energy trade-off

The inclusion of DHEAS in the ASI is not redundant but essential for understanding the metabolic cost of resilience. Chronic stress forces the organism into a “catabolic dominance” to prioritize immediate survival (mediated by cortisol) over long-term tissue repair (mediated by DHEAS).

DHEAS functions as a critical neuroprotective and immunomodulatory buffer, antagonizing the suppressive effects of cortisol on Natural Killer (NK) cells and Th1-mediated immunity (17, 19). A persistent decline in DHEAS, often seen in the ASI profiles of chronically stressed individuals, signifies an anabolic collapse. This state may reflect reduced anabolic buffering capacity associated with chronic stress.

### 6.3 Chronodisruption and the reprogramming of the endocrine network

The shift from a rhythmic, steep cortisol slope to a “flattened” profile represents more than just a loss of timing; it indicates a systemic reprogramming. As proposed by Straub et al. (29), chronic inflammatory diseases can be viewed as energy-deficiency syndromes. In this state, the immune system “usurps” the endocrine system to redirect energy substrates toward inflammatory sites.

The flattened diurnal slope and the “Adrenal Paradox” (where sex steroids may be elevated despite low adrenal precursors) suggest that peripheral tissues and immune cells have taken autonomous control over steroid metabolism, bypassing the central HPA regulation (29). The ASI protocol provides one possible approach for characterizing circadian endocrine organization in ambulatory settings.

In conclusion, the ASI may be considered not as a test for an imaginary “Adrenal Exhaustion”, but as a hypothesis-driven assessment of the organism’s inflammatory resilience. The ASI is therefore proposed here primarily as a functional interpretive framework and hypothesis-generating model, rather than as a definitive diagnostic test within conventional clinical endocrinology. Importantly, the present work does not seek to advocate for immediate clinical implementation of the ASI as a diagnostic tool. Rather, it aims to provide a mechanistic and quantitative framework to guide future experimental and longitudinal research designed to test its physiological and clinical relevance. It may help to identify the transition point at which the HPA axis shifts from adaptive regulation toward chronic inflammatory demand.

### 6.4 Conceptual and research implications

The framework proposed here is intended to support testable research questions regarding endocrine–immune coordination, rather than to prescribe clinical management or to argue for diagnostic adoption. In this view, ASI-derived descriptors may be used to operationalize candidate domains for empirical study.

First, profiles characterized by low or flattened salivary cortisol despite normal systemic measures motivate mechanistic investigation into peripheral hormone handling and delivery, including neutrophil-related pathways proposed in this manuscript. Second, a high integrated cortisol exposure relative to DHEAS suggests a catabolic–anabolic imbalance that can be examined in relation to inflammatory markers, functional outcomes, and aging-related phenotypes. Third, reduced circadian amplitude or altered temporal organization motivates longitudinal and experimental designs targeting circadian alignment to test whether modifying sleep–wake and light–dark patterns changes ASI descriptors and related physiological measures.

Crucially, these domains should be treated as hypotheses. Future studies should prioritize repeated-measures designs to quantify within-person variability, determine trait-like stability versus state dependence, and establish whether DHEAS co-varies with day-to-day changes in cortisol dynamics. These steps are prerequisites for evaluating any potential translational relevance of ASI-based phenotyping.

## 7 Conclusions: toward a new paradigm of endocrine resilience

The transition from the clinically vague concept of “adrenal fatigue” to the rigorous model of HPA-Immune Chronodisruption represents more than a semantic shift; it is a necessary evolution in the management of chronic inflammatory diseases. As we propose, the Adrenal Stress Index (ASI) is not a measure of glandular failure, but a functional proxy for the organism’s ability to maintain allostatic homeostasis under persistent demand.

Our hypothesis proposes a possible mechanistic link in the current understanding of glucocorticoid dynamics by identifying the neutrophil-CBG-elastase axis as a potential site of failure in chronic stress. This model is presented as a testable hypothesis intended to stimulate further experimental and clinical investigation. By recognizing that the bioavailability of cortisol is dependent on a finite pool of effector-cell granules, this framework may help explain the “silent” inflammation seen in patients with normal serum cortisol but flattened salivary profiles. In this light, the ASI may be considered as a functional interpretive tool for acquired local glucocorticoid resistance, offering a functional window into the tissue-level availability of the hormone that systemic blood tests cannot provide.

Furthermore, the integration of DHEAS as a metabolic buffer and the analysis of circadian rhythmicity move the focus from hormone levels to hormone timing and balance. The loss of the cortisol slope and the collapse of the anabolic reserve (DHEAS) are not just symptoms of stress; they are drivers of further systemic decline, contributing to a self–perpetuating cycle of metabolic and immune weathering.

Future research should also investigate the stability and variability of these quantitative descriptors across repeated measurements in the same individuals, in order to distinguish state-dependent fluctuations from trait-level regulatory patterns. If our hypothesis is correct, the restoration of HPA rhythmicity and the protection of the “neutrophil pool” could represent potential targets for future investigation.

These observations support the value of integrative approaches to endocrine physiology. We must now embrace a systemic, chronobiological approach to endocrinology, where the ASI may serve as a functional observational tool, documenting the organism’s complex, and often failing, struggle to sustain the energetic and inflammatory costs of life. Future research should also consider the potential influence of Early Life Stress (ELS), which is known to shape adult HPA axis regulation and inflammatory physiology, and may therefore contribute to interindividual variability in ASI profiles.

## Data Availability

The original contributions presented in the study are included in the article/supplementary material. Further inquiries can be directed to the corresponding author.
